# Primary Research on Correlation between Erectile Function Assessment through International Index of Erectile Function Score and Nocturnal Penile Tumescence and Rigidity Measurements in Men with Erectile Dysfunction

**DOI:** 10.12688/f1000research.149499.2

**Published:** 2025-07-30

**Authors:** Roberto Bagaskara Indy Christanto, Cindy Wijaya, Ponco Birowo, Widi Atmoko, Gerhard Reinaldi Situmorang, Ashok Agarwal, Rupin Shah

**Affiliations:** 1 Department of Urology, Rumah Sakit Dr Cipto Mangunkusumo, Faculty of Medicine, Universitas Indonesia, Central Jakarta, Jakarta, 10430, Indonesia; 2 Cleveland Clinic, Cleveland, Ohio, USA; 3 Lilavati Hospital and Research Centre, Mumbai, Maharashtra, India

**Keywords:** IIEF-5, Erectile Dysfunction, Nocturnal Penile Tumescence and Rigidity, Diagnosis

## Abstract

**Introduction:**

Erectile dysfunction (ED) affects approximately 40% of men; however, the true prevalence remains uncertain owing to various factors. Diagnosing ED is challenging, and tools like the International Index of Erectile Function (IIEF) and its shorter version, the IIEF-5, are commonly used to assess its severity. Although nocturnal penile tumescence and rigidity (NPTR) monitoring, as an objective test, can help diagnose ED, it is complex and not economical. Therefore, this study aimed to compare NPTR with the IIEF to assess the IIEF’s potential as a cost-effective diagnostic tool for ED.

**Methods:**

A correlation analysis study was performed on 139 men with ED between August 2017 and March 2023 who had undergone NPTR assessment in Jakarta, Indonesia. ED was assessed using detailed evaluations and IIEF-5 questionnaires. NPTR data was collected using a Rigiscan ^®^ device. Serum testosterone, total cholesterol, HbA1c, and vitamin D-25(OH) from blood samples were also evaluated. Bivariate analysis was used to explore the correlations between IIEF-5 scores, NPTR measurements, and blood parameters.

**Results:**

In total, 139 men with ED (median age: 42 years) were included. The median IIEF-5 score was 11, and comorbidities included dyslipidemia (20%) and diabetes (12%). There are significant correlations between IIEF scores and NPTR variables (number of erections recorded, base tumescence increment, average base tumescence, and duration of base erection with ˃ 60% rigidity). Additionally, significant negative correlations were observed between HbA1c levels and several NPTR variables, including the number and quality of nocturnal erections. Significant correlations were also found between HbA1c and various NPTR variables.

**Conclusion:**

This study underscores the value of subjective questionnaires such as the IIEF-5 in diagnosing ED, especially in the absence of advanced tests like the NPTR assessment. We found correlations between IIEF scores and nocturnal erection frequency, as well as specific erection characteristics. Our findings highlight the importance of a personalized approach to ED diagnosis. We also observed that higher HbA1c levels were associated with reduced nocturnal erection quality, highlighting the metabolic influence on erectile function. Although IIEF aids in cost-effective assessments, it should not replace objective testing.

## Introduction

Erectile dysfunction (ED), difficulty in obtaining or maintaining an erection to complete sexual intercourse, is found in approximately 40% of men. ^1^ Because of the existing cultural factors, reporting bias, and patient considerations, such as shame and embarrassment, the true prevalence of ED is yet to be determined. ED may occur due to various etiologies and is often multifactorial. ^2^ Establishing ED diagnosis and differentiating between psychogenic and organic ED is imperative for physicians. ^3, 4^

Diagnostic approaches in ED remain challenging, as information obtained from patient history and physical examination may not suffice. Supplemental examinations may be needed, and non-invasive tests are generally preferred. Therefore, the scoring system and questionnaire are vital for assessing ED severity and treatment progress. ^5^

The International Index of Erectile Function (IIEF) is a widely accepted patient-reported outcome measure (PROM) to evaluate ED. The IIEF aims to assess several domains of male sexual function, consisting of erectile function, orgasmic function, sexual desire, intercourse satisfaction, and overall satisfaction. ^6^ A shorter version of the five questions, known as the IIEF-5, was developed to assess patients’ erectile function and sexual intercourse satisfaction. The lower the IIEF-5 scores, the higher the severity of ED. ^7^

Nocturnal penile tumescence and rigidity (NPTR) monitoring with RigiScan device has been considered one of the most reliable methods to differentiate psychogenic erectile dysfunction (pED) from organic ED and to diagnose ED in general. ^8^ Nonetheless, NPTR monitoring has shortcomings, as previously reported. ^9, 10^ Furthermore, the main disadvantages of NPTR assessment include its high cost, intricate nature, and limited ability to yield consistent results.

Other means of ED diagnostics incorporate laboratory tests such as hypothalamic-pituitary-gonadal axis evaluation, including testosterone, luteinizing hormone (LH), follicle-stimulating hormone (FSH), lipid profile, blood glucose, and thyroid function tests. ^11^ However, as non-invasive and cost-efficient diagnostic evaluations are preferred, the IIEF could be an alternative diagnostic tool to assess Emerging evidence also suggests that several metabolic parameters, such as vitamin D, serum cholesterol, and glycated hemoglobin (HbA1c), may influence erectile function. Studies have found that low vitamin D levels, high cholesterol, and poor glycemic control are independently associated with ED severity. ^12^^,^^13^ Therefore, this study aimed to compare the parameters of NPTR testing with the domain score of the IIEF to determine the sufficiency of the IIEF in diagnosing ED. We also aimed to explore the correlation between erectile dysfunction and selected blood parameters.

## Methods

This was a correlation analysis study to determine the correlation between the IIEF-5 questionnaire and NPTR parameters. The data were collected through a database.

A total of 139 men presenting with ED between August 2017 and March 2023 who had undergone NPTR assessment were included in the study. The inclusion criteria were complaints of ED for at least 3 months without medical treatment. Men with depression, anxiety, and psychological problems, or those on antidepressant, antipsychotic, or anxiolytic medications, were excluded. Men with poor quality of sleep were also excluded from the study. All the patients were evaluated for complete medical histories, sexual histories, and physical examinations. The IIEF-5 questionnaire was used to evaluate the presence and severity of ED. The IIEF-5 contains five questions on erectile function and intercourse satisfaction. ^7^ This questionnaire is a non-proprietary tool that is widely used in clinical practice, including in our hospital, as a reliable and routine method for assessing erectile dysfunction severity.

Ethical approval was obtained from the Health Research Ethics Committee, Faculty of Medicine, Universitas Indonesia, and Cipto Mangunkusumo National General Hospital (No: KET-1624/UN2.F1/ETIK/PPM.00.02/2023), covering the use of patient data recorded in routine clinical care prior to study initiation. Study was conducted per the Declaration of Helsinki. As this study involved correlation analysis of de-identified medical records, the ethics committee waived the requirement for individual informed consent. All patient data used in this study were collected as part of routine standard care, and no identifying information was included in the analysis to ensure confidentiality.

### Nocturnal Penile Tumescence and Rigidity (NPTR)

NPTR was evaluated using the Rigiscan ^®^ device with Rigiscan Plus software (Timm Medical Technologies Inc., USA). The data collected from the NPTR examinations included the number of erections recorded, number of erections with normal tip duration, number of erections with normal tip tumescence, number of erections with adequate tip criteria, tip tumescence increment, average tip rigidity, duration of tip with > 60% rigidity, number of erections with normal base duration, number of erections with normal base tumescence, number of erections with adequate base criteria, base tumescence increment, average base rigidity, and duration of base with > 60% rigidity. Blood samples were collected before the NPTR examination and analyzed for total cholesterol, HbA1c, vitamin D-25(OH), and testosterone.

### Software availability

The Rigiscan device operates with the proprietary Rigiscan Plus software, which is included with the purchase of the device from Timm Medical Technologies Inc., USA. This software is essential for collecting and analyzing Nocturnal Penile Tumescence and Rigidity (NPTR) data. As this software is proprietary and bundled with the device, no free or open-source alternatives are available for equivalent functionality.

The Rigiscan device and its software are widely used in clinical practice as standard tools for erectile dysfunction assessment, and their use in this study complies with institutional clinical protocols.

### Statistical analysis

All statistical analyses were performed using SPSS 27.0 software (IBM, Armonk, NY, USA). All continuous data are presented as meaminus SD for normal distributions and medians (min-max) for data, not within the normal distribution. The normal distribution of variables was analyzed using the Kolmogorov–Smirnoff sample test. Table 1 summarizes the patients’ characteristics. The correlation between the IIEF-5 score and NPTR measurements was performed using Pearson bivariate analysis for normally distributed data or Spearman bivariate analysis for skewed distribution. A p<0.05 indicated statistical significance. Further bivariate analysis was performed to analyze the correlation between blood parameters and NPTR measurement.

**Table 1.** Patients’ characteristics (n=139).

Variable	Median (range) or N
Age (in years)	42 (19–76)
IIEF-5 score	11 (0–24)
Dyslipidemia	27
Type-2 DM	
Prediabetic	65
Diabetic	16

## Results

A total of 139 men with ED were included in the study. The median patient age was 42 years. The IIEF-5 scores ranged between 0 and 24, with a median of 11. Comorbidities included dyslipidemia in 27 patients (20%), prediabetes in 65 patients (47%), and diabetes in 16 patients (12%).

The Kolmogorov–Smirnov normality test showed that all numeric variables of NPTR measurements did not have a normal data distribution ( Table 2). Pearson’s correlation for the IIEF scores vs. NPTR variables showed significant correlations with the number of erections recorded *r*=0.188 (95% confidence interval (CI), 0.017–0.348; *P*=0.026), base tumescence increment *r*=0.198 (95% CI, 0.027–0.357; *P*=0.020), average base tumescence *r*=0.185 (95% CI, 0.014–0.346; *P*=0.029), and duration of base erection with more than 60% rigidity *r*=0.198 (95% CI, 0.027–0.357; *P*=0.020). No correlations were observed between the IIEF scores and other NPTR variables ( Table 3).

**Table 2.** Nocturnal penile tumescence and rigidity (NPTR) measurements (n=139).

Variable	Median (range)
Number of erection(s) recorded	3 (0–10)
Number of erection(s) with normal tip duration	0 (0–4)
Number of erection(s) with normal tip tumescence	0 (0–6)
Number of erection(s) with adequate tip criteria	0 (0–4)
Δ Tip Tumescence	0.9 (0.0–3.4)
Average tip rigidity	38.0 (0.0e85.0)
Duration of tip with > 60% rigidity	660 (0–10530)
Number of erection(s) with normal base duration	2 (0–6)
Number of erection(s) with normal base tumescence	0 (0–5)
Number of erection(s) with adequate base criteria	0 (0–3)
Δ Base Tumescence	1.9 (0.0–3.6)
Average base rigidity	55.5 (0.0–100.0)
Duration of base with > 60% rigidity	2280 (0–15480)

**Table 3.** IIEF correlations with nocturnal penile tumescence and rigidity (NPTR) measurements (n=139).

Variables	r	95% CI	*P*-value
Number of erection(s) recorded	0.188*	0.017–0.348	0.026
Number of erection(s) with normal tip duration	0.004	-0.168–0.175	0.967
Number of erection(s) with normal tip tumescence	0.115	-0.058–0.281	0.179
Number of erection(s) with adequate tip criteria	0.162	-0.010–0.324	0.057
Δ Tip Tumescence	0.134	-0.039–0.298	0.117
Average tip tumescence	0.184	0.013–0.344	0.030
Average tip rigidity	0.093	-0.079–0.260	0.275
Duration of tip with > 60% rigidity	0.111	-0.061–2.77	0.192
Number of erection(s) with normal base duration	0.151	-0.021–0.314	0.077
Number of erection(s) with normal base tumescence	0.083	-0.090–0.250	0.333
Number of erection(s) with adequate base criteria	0.064	-0.108–0.233	0.452
Δ Base Tumescence	0.198*	0.027–0.357	0.020
Average base tumescence	0.185*	0.014–0.346	0.029
Average base rigidity	0.163	-0.008–0.326	0.055
Duration of base with > 60% rigidity	0.198*	0.027e0.357	0.020

Pearson’s correlation for HbA1c vs. NPTR variables showed significant correlations with the number of erections recorded *r*=-0.280 (95% CI; *P*<0.01), number of erections with normal tip duration *r*=-0.238 (95% CI; *P*<0.05), tip tumescence increment *r*=-0.269 (95% CI; *P*<0.05), average tip rigidity *r*=-0.336 (95% CI; *P*<0.01), duration of tip erection with > 60% rigidity *r*=-0.277 (95% CI; *P*<0.01), number of erections with normal base duration *r*=-0.249 (95% CI; *P*<0.05), and duration of base erection with >60% rigidity *r*=-0.262 (95% CI; *P*<0.05). No significant correlations were observed between NPTR variables and other blood parameters (total cholesterol, vitamin D 25(OH), and testosterone) ( Table 4).

**Table 4.** Correlation between nocturnal penile tumescence and rigidity (NPTR) measurements and blood parameters.

Variables	Total cholesterol (n=97)	HbA1c (n=85)	Vitamin D-25(OH) (n=97)	Testosterone (n=96)
Number of erection(s) recorded	0.075	**-0.280** **	0.051	0.067
Number of erection(s) with normal tip duration	0.024	**-0.238** *	-0.040	-0.096
Number of erection(s) with normal tip tumescence	0.092	-0.006	-0.110	-0.024
Number of erection(s) with adequate tip criteria	0.042	0.019	-0.095	-0.177
Δ Tip Tumescence	-0.056	**-0.269** *	-0.022	0.009
Average tip tumescence	-0.066	-0.203	-0.039	-0.073
Average tip rigidity	-0.020	**-0.336** **	0.009	-0.032
Duration of tip with > 60% rigidity	-0.003	**-0.277** **	0.040	-0.049
Number of erection(s) with normal base duration	0.099	**-0.249** *	0.049	-0.119
Number of erection(s) with normal base tumescence	0.075	-0.171	-0.048	-0.104
Number of erection(s) with adequate base criteria	0.116	-0.172	-0.051	-0.103
Δ Base tumescence	0.065	-0.140	-0.028	-0.037
Average base tumescence	092	-0.055	-0.063	-0.128
Average base rigidity	-0.064	-0.168	0.143	-0.030
Duration of base with > 60% rigidity	0.136	**-0.262** *	0.059	-0.097

* p-value<0.05.

** p-value<0.01.

## Discussion

### Correlation between IIEF-5 and NPTR parameters

To our knowledge, this study was the first to explore the association between the IIEF-5 score and specific components of nocturnal erection using NPTR or NPT measurements. Although previous studies have examined the correlation between nocturnal erections and IIEF-5 questionnaire scores, serving as a clinical measure of ED, these studies have primarily categorized nocturnal erections as either “adequate” or “inadequate” and compared them with the IIEF-5 questionnaire scores. The components of nocturnal erections encompass various aspects, including the number of erections, the quality of erections, and aspects related to both the tip and the base of the erection. The present study aimed to compare and correlate these individual aspects with IIEF-5 questionnaire scores.

### Overview of NPTR physiology and assessment

Spontaneous nighttime penile erections, initially observed by Halverson et al. in infants during sleep in 1940 and confirmed by subsequent studies, are part of the normal NPTR phenomenon. ^14^ They involve 3–6 episodes of tumescence during sleep, including at least one instance with a tip rigidity exceeding 60%, lasting 1–15 min over 8 h. ^15^ These regular NPTR occurrences indicate a healthy vascular and neural supply to the penis and the structural integrity of penile components. NPTR assessment employs seven methods, with the Rigiscan device introduced in 1985. ^16^ Rigiscan device provides continuous and quantitative measurements of penile circumference and rigidity, helping distinguish between physiological ED and organic cases of ED. This differentiation relies on the assumption that psychological factors do not substantially impact nighttime erectile activity. ^8^

### Strengths and limitations of NPTR testing

The application of NPT relies on the premise that to generate a nocturnal erection, both the corticospinal nerve signals to the penis and the vascular responsiveness of penile tissue to these signals must remain functional. Nevertheless, achieving an erection during sexual activity also necessitates proper responsiveness to sensory cues. Notably, the inability to assess any deficits in afferent signals originating from the penis is a potential limitation of NPT. ^17^

Consequently, NPTR has been subjected to validation procedures recently. The evolution of the Rigiscan Plus software has led to a standardized approach to NPTR examinations, thereby enhancing sensitivity and specificity and allowing for the precise identification of erections possessing the requisite rigidity and duration for engaging in sexual intercourse. Empirical studies have reported sensitivities ranging from 42% to 85% and specificities ranging from 93% to 100%. ^17– 19^ In another investigation, the study examined the sensitivity, specificity, positive predictive value, and accuracy rate of Rigiscan device monitoring in differentiating organic and pED. ^20^ These assessments were made in the context of a comprehensive diagnostic approach incorporating advanced techniques, and the results indicated values of 81%, 82%, 89%, and 81%, respectively, for NPTR measurements using the Rigiscan device. ^20^

However, the utilization of NPTR has several limitations, as highlighted in previous studies. One study that employed NPT with the Minnesota Multiphasic Personality Inventory for diagnosing primary organic or primary psychogenic ED reported a misclassification rate of 63%. ^9^ Certain specific populations further complicate the accuracy of NPT assessment, with a decreased diagnostic accuracy observed in men experiencing depression. ^10^ Additionally, the absence of universally accepted normative data for interpreting NPTR readings makes it challenging to reproduce the assessments. ^15^

The utilization of NPTR assessment presents a substantial drawback owing to its cost, complexity, and relatively low reproducibility. Moreover, it is worth considering that endocrine, vascular, and neurological diagnostic techniques offer cost-effective alternatives and can identify organic causes of ED to resolve ED issues. ^21^ These limitations prompt us to question whether standardized questionnaires, such as the IIEF-5, may suffice as more cost-effective diagnostic measures in conjunction with laboratory investigations. Consequently, our primary aim was to explore the correlation between the IIEF-5 questionnaires and specific parameters within NPTR measurements.

### Diagnostic value and limitations of IIEF-5

The IIEF questionnaire was initially created not for diagnostic purposes but as a tool to track treatment outcomes over time. ^17^ Moreover, IIEF and similar self-assessment questionnaires cannot discriminate among different etiologies of ED. ^22, 23^ Nevertheless, examining its association with objective assessments like NPTR is intriguing. Kassouf et al. examined the diagnostic utility of the IIEF-5 in identifying the vascular causes and severity of ED, comparing it with pharmacological testing and duplex Doppler ultrasonography. ^24^ Their findings showed no significant differences in IIEF scores among patients with normal vascular responses, arterial insufficiency, or venous leakage.

### Correlation between IIEF-5 and NPTR findings

The median number of erections recorded in our study was three, which is consistent with previous studies that reported a similar number of erection episodes in patients without diabetes. ^25^ Our findings revealed a significant correlation between the IIEF-5 questionnaire and the number of recorded nocturnal erections. These findings bolster the hypothesis that a reduced number of nocturnal erections may manifest as subjective erectile symptoms.

Our findings indicate that the base aspects of penile erections are the most strongly correlated with the IIEF-5 score, with no significant correlation observed for tip erections. In contrast, Lee et al. (2021) analyzed the potential link between lower urinary tract symptoms (LUTS) associated with benign prostatic hyperplasia (BPH) and erectile function in eugonadal men and demonstrated that the rigidity activity unit in the penile tip emerged as the most statistically significant parameter in its association with the IIEF score. ^26^

Both tip and base erections are essential in assessing erectile function. However, based on a cross-sectional study on the effect of prolactin on penile erection, the penile base may be more meaningful than the penile tip in assessing the effect of prolactin on erectile function. ^27^ Another cross-sectional study on the effect of estradiol on penile erection found no apparent relationship between erection time at the penile tip and estradiol levels. However, a negative correlation was observed between base erection time and estradiol levels. ^28^

### Association with metabolic and hormonal parameters

In the context of blood parameters, this study determined that only HbA1c levels displayed a statistically significant correlation with several NPTR parameters. Several studies have investigated the relationship between diabetes and nocturnal penile tumescence (NPT); NPT test results were abnormal in sexually functional men with diabetes. ^29^ 10 men with diabetes who reported normal daytime sexual function with four nights of polysomnography, including NPT assessment, had significantly diminished NPT profiles compared to an age-matched, nondiabetic, healthy control group. ^30^ Anwar et al. revealed that in ED and diabetes, men with diabetes experiencing severe ED tended to be older and experience more challenges maintaining proper glycemic control. ^31^

No significant correlation was observed between NPTR variables and other blood parameters (total cholesterol, vitamin D-25(OH), and testosterone). The two main types of cholesterol are high-density lipoprotein (HDL) and low-density lipoprotein (LDL). Elevated levels of LDL can contribute to the development of ED, whereas HDL cholesterol improves penile erectile function. ^32^ Therefore, the relationship between cholesterol and nocturnal erections may depend on the measured cholesterol type. Furthermore, a study has found that statin drugs improve erectile function in men with hypercholesterolemia. ^33^ Therefore, statin drugs may mask any potential correlation between cholesterol and nocturnal erections.

Low serum 25-hydroxyvitamin D (25(OH)D) levels were associated with an increased risk of ED in older adults with moderate to severe lower urinary tract symptoms. ^34^ Furthermore, a study by Dumbraveanu et al. investigated the correlations of clinical and biochemical indices of vitamin D with ED. ^35^ The study found that vitamin D level reduction, concomitantly with decreased testosterone and increased cholesterol, contributed to the development and maintenance of ED. Further research is needed to validate this association.

A study investigating the relationship between sleep-related erections and testosterone levels in men found that the serum testosterone threshold for sleep-related erections was lower than the low end of the standard laboratory male range at approximately 200 ng/dL. ^36^ The study also found that participants with higher testosterone serum levels showed higher values for some erectile parameters than those with serum testosterone between 100 and 199 ng/dL, without any significant difference among the groups with testosterone serum levels in the normal range. ^36^ Although some studies suggest a correlation between NPT and testosterone, other studies demonstrated no significant correlation. A review article on the relationship between testosterone and sleep-related erections reported that men with androgen deficiency may have normal NPT, and sleep-related erections increased in response to testosterone administration. ^37^ A prospective cross-sectional pilot study found that testosterone levels were weakly associated with penile rigidity and disappeared when associated with metabolic syndrome. ^38^

### Study limitations

The present study had several limitations. First, the study was not population-based. The patients included in the study were individuals seeking treatment at a urologist’s office who reported erectile ED and underwent NPTR testing. Therefore, the current study was prone to selection bias. Second, not all patients were assessed for essential laboratory exams such as HbA1c, cholesterol, testosterone, and vitamin D-25(OH). Another aspect to consider is the use of single NPTR testing in our study, which may not have accurately evaluated nocturnal erectile capacity. Consecutive nocturnal measurements are widely accepted to differentiate pED from organic ED. However, only a single NPTR test was performed in this study to prioritize patient comfort. Moreover, the correlation study design might have introduced biases, and the sample size, constrained by specific criteria and exclusions (e.g., depression, anxiety, poor sleep quality), may have impacted the generalizability of the results. Additionally, reliance on secondary data and potential variations in recording practices might have affected the reliability of the results. We acknowledge that psychosocial and interpersonal aspects were not thoroughly evaluated in this study and should be considered in future research.

## Conclusion

Despite its limitations, this study provides insights into IIEF-5 and NPTR correlations within a specific cohort. Current research suggests that subjective questionnaires, including the IIEF-5, can be utilized in the diagnosis of ED, particularly in settings where advanced diagnostic tests, such as the NPTR assessment, are not available. We observed a correlation between IIEF questionnaire scores and the frequency of nocturnal erections, as well as the tip aspects of these nocturnal erections. Additionally, we identified a significant correlation between HbA1c levels and nocturnal erections. The method used to diagnose ED should be personalized and multidisciplinary. The primary objective should be to conduct a thorough assessment of erectile function, and NPTR assessment, which was used to monitor NPT, remains a valuable diagnostic tool for ED. Although the IIEF can facilitate cost-effective diagnoses, it should not be considered as a substitute for objective testing.

## Ethical approval

Ethical approval for this study was obtained from the Health Research Ethics Committee, Faculty of Medicine, Universitas Indonesia, and Cipto Mangunkusumo National General Hospital. The approval number is KET-1624/UN2.F1/ETIK/PPM.00.02/2023, and the approval was granted on November 17, 2023.

The study was conducted in accordance with the ethical principles outlined in the Declaration of Helsinki. As the study involved correlation analysis of anonymized medical records collected during routine care, the ethics committee waived the requirement for individual informed consent. To ensure confidentiality, all patient data were de-identified prior to analysis.

## Consent

This study utilized secondary data from patient medical records collected during routine clinical care. Ethical approval was obtained from the Health Research Ethics Committee, Faculty of Medicine, Universitas Indonesia, and Cipto Mangunkusumo National General Hospital (Approval No: KET-1624/UN2.F1/ETIK/PPM.00.02/2023). The committee waived the requirement for individual informed consent as the data were anonymized and de-identified prior to analysis to ensure patient confidentiality. No additional data collection or interventions were performed as part of this study, and all analyses were conducted in compliance with the Declaration of Helsinki.

## Data Availability

All raw data underlying the findings of this study, prior to any data analysis, have been made publicly available through the Open Science Framework (OSF). The data can be accessed through Open Science Framework: Correlation between Erectile Function Assessment through International Index of Erectile Function Score and Nocturnal Penile Tumescence and Rigidity Measurements in Men with Erectile Dysfunction. OSF | Correlation between Erectile Function Assessment through International Index of Erectile Function Score and Nocturnal Penile Tumescence and Rigidity Measurements in Men with Erectile Dysfunction. ^39^ Data are available under the terms of the Creative Commons Zero “No rights reserved” data waiver (CC0 1.0 Public domain dedication). All supplementary materials and documents used in this study have been made publicly available through the Open Science Framework (OSF). The extended data include the International Index of Erectile Function (IIEF) questionnaires, as well as other supporting materials. The extended data can be accessed through Open Science Framework: Correlation between Erectile Function Assessment through International Index of Erectile Function Score and Nocturnal Penile Tumescence and Rigidity Measurements in Men with Erectile Dysfunction. https://www.doi.org/10.17605/OSF.IO/XFWYQ. ^39^ Data are available under the terms of the Creative Commons Zero “No rights reserved” data waiver (CC0 1.0 Public domain dedication).
