# Supplementary material for: Primary Research on Correlation between Erectile Function Assessment through International Index of Erectile Function Score and Nocturnal Penile Tumescence and Rigidity Measurements in Men with Erectile Dysfunction
Source: F1000Res. 2025 Jul 30;14:142. Originally published 2025 Jan 28. [Version 2] doi: 10.12688/f1000research.149499.2 (PMC12365589; doi:10.12688/f1000research.149499.2)
Supplement: Peer review history

# Reviewer report: Reviewer response for version 2

**DOI:** 10.5256/f1000research.184430.r401594

**Published:** 2025-08-19

**Authors:** Syah Mirsya Warli

**Affiliations:** 1 Universitas Sumatera Utara, Medan, Indonesia

I have reviewed the revised manuscript and have no further comments from my side. Thank you for the opportunity to review.

Is the work clearly and accurately presented and does it cite the current literature?

Yes

If applicable, is the statistical analysis and its interpretation appropriate?

Partly

Are all the source data underlying the results available to ensure full reproducibility?

Partly

Is the study design appropriate and is the work technically sound?

No

Are the conclusions drawn adequately supported by the results?

Partly

Are sufficient details of methods and analysis provided to allow replication by others?

Yes

Reviewer Expertise:

Urology, Oncology, Andrology

I confirm that I have read this submission and believe that I have an appropriate level of expertise to confirm that it is of an acceptable scientific standard.

# Reviewer report: Reviewer response for version 2

**DOI:** 10.5256/f1000research.184430.r401595

**Published:** 2025-08-04

**Authors:** Hong-Jun Li

**Affiliations:** 1 Department of Urology, Peking Union Medical College, Peking Union Medical College Hospital, Chinese Academy of Medical Sciences, Beijing, China

I am very happy to read the revised paper, because all my concerns are revised, adjusted and supplemented.

 Another suggestion is as follow:

 It is not wise to state that “To our knowledge, this study was the first to explore the association between the IIEF-5 score and specific components of nocturnal erection using NPTR or NPT measurements”, and also the expression is not much valuable. Therefore, from the opinion of modest and objectiveness, I suggest delete the sentence. Actually, related researches are existed.

Is the work clearly and accurately presented and does it cite the current literature?

Yes

If applicable, is the statistical analysis and its interpretation appropriate?

I cannot comment. A qualified statistician is required.

Are all the source data underlying the results available to ensure full reproducibility?

Partly

Is the study design appropriate and is the work technically sound?

Partly

Are the conclusions drawn adequately supported by the results?

Partly

Are sufficient details of methods and analysis provided to allow replication by others?

Partly

Reviewer Expertise:

basic and clinical research on andrology

I confirm that I have read this submission and believe that I have an appropriate level of expertise to confirm that it is of an acceptable scientific standard.

# Reviewer report: Reviewer response for version 1

**DOI:** 10.5256/f1000research.163969.r376089

**Published:** 2025-04-11

**Authors:** Syah Mirsya Warli

**Affiliations:** 1 Universitas Sumatera Utara, Medan, Indonesia

First of all, I would like to comment about the idea and the title itself. All of us know that IIEF-5 Questionnaire is meant to be a screening tools, not a diagnostic tool. Meanwhile, NPTR is an objective diagnostic tool which widely known as the gold standard for Erectile Dysfunction. Finding a correlation between those two might be unwise. However, I understand that the author has the idea of promoting IIEF-5 as a diagnostic tool in a rural area which NPTR might not be available

 Secondly, I would like to comment about the method of this study. The author wrote that this study is a retrospective cohort study. As we know, cohort is a a type of observational study that follows a group of individuals (a cohort) with a shared characteristic over time to examine how certain factors or exposures affect their health outcomes, which is not the case in this study. The end result of Cohort study should be Relative Risk, while this study is trying to find a correlation between two diagnostic tools. I suggest to change the term in methods section from A retrospective cohort study to a correlation analysis study. In addition, I would like to appreciate the author on using the right statistical tools, which is Kolmogorov Smirnoff analysis on determining the data distribution. In this study, we found that the data is non-parametric. Therefore, Spearman analysis is used in this study. The author also presents the data in median instead of mean, which is the correct way to present a non-parametric data.

 Thirdly, I also found an unusual thing in this manuscript. This study aim is to find a correlation two diagnostic tools. I do not see the importance of performing evaluation on serum testosterone, total cholesterol, HbA1c, and vitamin D-25(OH). The title, the method, the result and the conclusion of this study is scrambled. If you want to add those data in this manuscript, the author should add the study aims in the introduction. I suggest the author to add the study aims as this study would like to investigate the correlation of erectile dysfunction with several variable such as serum testosterone, total cholesterol, HbA1c, and vitamin D-25(OH). The other options would be to delete all of those from this manuscript.

Is the work clearly and accurately presented and does it cite the current literature?

Yes

If applicable, is the statistical analysis and its interpretation appropriate?

Partly

Are all the source data underlying the results available to ensure full reproducibility?

Partly

Is the study design appropriate and is the work technically sound?

No

Are the conclusions drawn adequately supported by the results?

Partly

Are sufficient details of methods and analysis provided to allow replication by others?

Yes

Reviewer Expertise:

Urology, Oncology, Andrology

I confirm that I have read this submission and believe that I have an appropriate level of expertise to confirm that it is of an acceptable scientific standard, however I have significant reservations, as outlined above.

## References

[1] : Research Design: Cohort Studies. *Indian J Psychol Med* .2022;44(2) : 10.1177/02537176211073764 189-191 10.1177/02537176211073764 35655982 PMC9120971

# Response

**Published:** 2025-07-02

**Authors:** Ponco Birowo

**Affiliations:** Department of Urology Faculty of Medicine Universitas Indonesia/ CIpto Mangunkusumo Hospital, Indonesia

1. We clarified that the IIEF-5 should not replace NPTR but may serve as an initial tool in settings lacking advanced diagnostics. Added the sentence: "Although the IIEF aids in cost-effective assessments, it should not replace objective testing."

 2. We have rephrased our study design in the *Methods* section: The phrase "A retrospective cohort study" was replaced with "a correlation analysis study", as suggested.

 3. Regarding the relevance of the blood parameters, we have revised our *Introduction*: Added the sentence: "This study also aimed to explore the correlation between erectile dysfunction and selected blood parameters (serum testosterone, total cholesterol, HbA1c, and vitamin D-25(OH))." This aligns the aim with the presented data. We have also added a discussion section regarding the relevance of these parameters with erectile dysfunction.

 4. Regarding the mentions of genital and psychosocial examinations, We have added in the *Discussion*: "All subjects underwent a comprehensive physical examination, and no abnormalities nor defects of the genital organs were observed." "We acknowledge that psychosocial and interpersonal aspects were not thoroughly evaluated in this study and should be considered in future research."

# Reviewer report: Reviewer response for version 1

**DOI:** 10.5256/f1000research.163969.r376083

**Published:** 2025-04-08

**Authors:** Hong-Jun Li

**Affiliations:** 1 Department of Urology, Peking Union Medical College, Peking Union Medical College Hospital, Chinese Academy of Medical Sciences, Beijing, China

Due to the existing cultural factors, reporting bias, and patient considerations, diagnosing ED accurately is challenging. Both the International Index of Erectile Function (IIEF)-5 score and an objective test with nocturnal penile tumescence and rigidity (NPTR) monitoring are all used in routine diagnostic evaluation of men with Erectile Dysfunction. Of course, I accept that diagnosis of ED with NPTR is relatively complex and not economical. The authors tried to compare NPTR with the IIEF to assess the IIEF’ potentials as a cost-effective diagnostic tool for ED, and concluded correlations between IIEF scores and nocturnal erection frequency, as well as specific erection characteristics. The research was meaningful and interesting, but some problems should be considered before it can be accepted for indexing.

 My major concerns are as follows:

 The title does not indicate the specific type of article. It is recommended to add a few words before the title，that is “Primary Research on”. Primary Research on the Correlation between Erectile Function Assessment through International Index of Erectile Function Score and Nocturnal Penile Tumescence and Rigidity Measurements in Men with Erectile Dysfunction.

 Research design was simple and clear. No examination results were available for the genital area, including testicular volume and varicocele. In addition to that, the real challenge of ED diagnosis is mainly the patient's mental and psychological state, as well as their interpersonal relationships, particularly with sexual partners, which were not considered by the author. Mentioning these aspects in the introduction or discussion might provide a more comprehensive analysis. It would be even more perfect if it could be included in the study design.

 Was the author concerned about the NPTR test being affected by the patient's sleep conditions?

 How is the sample size determined? The sample size of this study (138 cases) is somewhat small, the age range of the enrolled patients was quite broad（19–76），and the duration of the practical research period (6 years) is relatively long. This makes it difficult to conduct detailed analysis and increases the likelihood that changes in lifestyle, environment, cognition, diagnostic methods, and criteria could affect the results and conclusions. For instance, testosterone levels are significantly related to age, with middle-aged and older men showing marked differences compared to younger adults. The small sample size makes it impossible to discuss testosterone issues by age group. Similar situations are widespread, affecting the reliability and objectivity of the various analyses presented in this article.

 It's strange that the abstract mentions 138 patients included in the method, but the results section shows 139 patients.

 It is well accepted that there are significant correlations between IIEF scores and NPTR variables. Therefore, the main results and conclusions for the paper are not very valuable, and the author should explore deeply to demonstrate the paper’s value.

 Conclusions in the abstract were not properly organized. Results in abstract could not cover the conclusions. In another way, some conclusions in abstract did not based on the results in abstract, such as last part sentences in abstract, that was “Our findings highlight the importance of a personalized approach to ED diagnosis. Although IIEF aids in cost effective assessments, it should not replace objective testing”.

 It is recommended to use subheadings in the discussion section to clearly display the topic of each paragraph, which helps with the organization and logical flow of the article.

Is the work clearly and accurately presented and does it cite the current literature?

Yes

If applicable, is the statistical analysis and its interpretation appropriate?

I cannot comment. A qualified statistician is required.

Are all the source data underlying the results available to ensure full reproducibility?

Partly

Is the study design appropriate and is the work technically sound?

Partly

Are the conclusions drawn adequately supported by the results?

Partly

Are sufficient details of methods and analysis provided to allow replication by others?

Partly

Reviewer Expertise:

basic and clinical research on andrology

I confirm that I have read this submission and believe that I have an appropriate level of expertise to confirm that it is of an acceptable scientific standard, however I have significant reservations, as outlined above.

# Response

**Published:** 2025-07-02

**Authors:** Ponco Birowo

**Affiliations:** Department of Urology Faculty of Medicine Universitas Indonesia/ CIpto Mangunkusumo Hospital, Indonesia

1. We have revises the title to our paper: " **Primary Research on the Correlation between Erectile Function Assessment through IIEF Score and NPTR Measurements…" ** as commented by the reviewer

 2. We have adjusted a few discrepancies that has been noted (number of subjects, results and conclusion in the abstract). We have also introduced subheadings in the discussion.

 3. A discussion on sample size has been added, explaining how the extended study period may introduce variability due to shifts in clinical practice, lifestyle, and diagnostic standards. We will also suggest stratified analysis in future research.

 4. Regarding the patient's sleeping conditions, we recognize that NPTR results are highly dependent on sleep quality. Prior to NPTR testing, each patient was given standardized instructions, including avoiding alcohol, sedatives, and strenuous physical activity 24 hours before the test, ensuring adequate sleep, and abstaining from sexual activity on the night of testing. These steps were intended to minimize external factors that could affect the results of nocturnal penile tumescence

 5. Regarding the examination for the genital area, we can confirm that all subjects underwent a comprehensive physical examination, and no abnormalities nor defects of the genital organs were observed. 

 6. We have also demonstrated the value of our paper through explanation tht our study now will serve as a pilot study in Indonesia, more comprehensive analysis will be advised for further enhancement of future studies
